# The Activation and Signaling Mechanisms of GPR56/ADGRG1 in Melanoma Cell

**DOI:** 10.3389/fonc.2018.00304

**Published:** 2018-08-08

**Authors:** Kuan-Yeh Huang, Hsi-Hsien Lin

**Affiliations:** ^1^Department of Microbiology and Immunology, College of Medicine, Chang Gung University, Taoyuan, Taiwan; ^2^Department of Anatomic Pathology, Chang Gung Memorial Hospital-Linkou, Taoyuan, Taiwan; ^3^Chang Gung Immunology Consortium, Chang Gung Memorial Hospital-Linkou, Taoyuan, Taiwan

**Keywords:** GPR56, melanoma, tumor progression, signaling pathways, adhesion GPCR

## Abstract

Adhesion G protein-coupled receptors (aGPCRs) constitute the second largest GPCR subfamily. GPR56/ADGRG1 is a member of the ADGRG subgroup of aGPCRs. Although GPR56 is best known for its pivotal role in the cerebral cortical development, it is also important for tumor progression. Numerous studies have revealed that GPR56 is expressed in various cancer types with a role in cancer cell adhesion, migration and metastasis. In a recent study, we found that the immobilized GPR56-specific CG4 antibody enhanced IL-6 production and migration ability of melanoma cells. In this review, we will summarize the current understanding of GPR56 function and discuss the activation and signaling mechanisms of GPR56 in melanoma cells.

## Adhesion GPCRs

G protein-coupled receptor (GPCR) superfamily is the largest receptor family in nature. Because of their signature seven-transmembrane (7TM) domain, GPCRs are also called the 7TM receptors (1). Based on their phylogenetic relationship GPCRs are further classified into five subfamilies: Glutamate, Rhodopsin, Adhesion, Frizzled, and Secretin (GRAFS) (1). Most of the adhesion GPCRs (aGPCRs) were found initially through the genomic approaches and were orphan receptors due to the lack of interacting ligands. A total of 33 aGPCR members were identified in human and were subdivided into eight groups based on the genetic and structural criteria (2, 3). In recent years, a number of aGPCRs have been implicated in various physiological functions, genetic disorders as well as tumorigenesis (3).

Structurally distinct from the canonical GPCRs, aGPCRs contain an unusually large extracellular domain (ECD) composed of a diverse range of cell adhesive protein motifs normally arranged in tandem repeats at the N-terminal half (4–6). This is followed by a signature auto-proteolytic protein fold of ~300 residues, called the GPCR auto-proteolysis inducing (GAIN) domain, which is immediately upstream of the 7TM domain (7, 8). A highly conserved GPCR proteolysis site (GPS) is embedded in the GAIN domain proximal to the first TM region (9). During aGPCR synthesis and maturation, auto-proteolytic processing at GPS takes place in the endoplasmic reticulum or in the Gogi complex (9). This novel post-translational proteolytic reaction dissects the aGPCR into two subunits, a long extracellular N-terminal fragment (NTF, α-subunit) and a 7TM C-terminal fragment (CTF, β-subunit). Interestingly, the cleaved NTF and CTF remain on the cell surface as a dual-component receptor complex via non-covalent interaction (9).

It is now well-accepted that a major activation mechanism of aGPCRs involves the binding of the aGPCR-NTF with its ligand(s) followed by activation and intracellular signaling of the corresponding CTF (10–12). Indeed, the so called tethered agonism whereby a newly exposed N-terminal agonist peptide of the CTF binds and activates the own CTF following the separation of the NTF from CTF has been shown for several aGPCRs (13, 14). In general, it is believed that the interaction of ligands/binding partners with the NTF in combination with mechanical disturbance lead to the detachment of the NTF-CTF complex and the subsequent manifestation of the Stachel agonist peptide for the activation of the own CTF. Nevertheless, the Stachel peptide-independent and GPS proteolysis-independent activation mechanisms have also been demonstrated for certain aGPCRs recently (15, 16). The activity and function of a given aGPCR hence is governed by multiple factors including the specific binding partners, receptor auto-proteolysis as well as micro-environmental changes.

## GPR56/ADGRG1

GPR56/ADGRG1, first reported by Liu et al. and Zendman et al. (17, 18), belongs to the ADGRG subfamily of aGPCRs (3). Mapped on the short arm of human chromosome 16 (16q13) with a total of 13 exons, GPR56 (also named TM7XN1) was differentially expressed in a panel of melanoma cell lines of which the highly metastatic ones were found to display a markedly reduced GPR56 expression. Expressional analysis of transcript message indicated that GPR56 was widely distributed in various tissues including brain, heart, testis and thyroid gland (17, 18). In addition to the GAIN domain, an additional structurally-defined domain was identified recently. The Pentraxin/Laminin/neurexin/sex-hormone-binding-globulin-Like (PLL) domain is located upstream of the GAIN domain at the most N-terminal region of the receptor (19). Herein, we will first review the biological and cellular functions of GPR56, followed by the identification of its binding partners/ligands, and finally the activation and signaling mechanisms of GPR56 in melanoma cells.

## Biological and cellular functions of GPR56

It is now known that GPR56 participates in a myriad of physiological processes. The best example is the link of GPR56 dysfunction to human developmental disorders (20–23). Indeed, GPR56 is critical for the frontal cortex development as loss-of-function GPR56 mutations causally lead to a rare but severe brain cortical malformation called bilateral frontoparietal polymicrogyria (BFPP) (20). The different types of BFPP-causing GPR56 mutations are widespread and include deletional, splicing, frameshift, and missense point mutations (20). Most interestingly at least 12 distinct missense point mutations were identified in BFPP patients, most of them located at the extracellular region of GPR56 (20, 24, 25). These disease-associated missense mutations were especially instrumental in revealing multiple distinct BFPP-causing mechanisms mediated by GPR56 (25–28). In mice, Gpr56 gene deficiency resulted in neuronal malformations in the cerebral cortex essentially recapitulating the BFPP phenotype (29). This study established firmly the role of GPR56 in regulating the integrity of pial basement membrane (BM) during cortical development. More recently, it was further shown that regional cerebral cortical patterning was tightly regulated by the GPR56 expression levels controlled in part by a specific cis-regulatory non-exonic element and generation of multiple alternative splicing forms (30).

In addition to the cortical development, GPR56 is also involved in oligodendrocyte development and CNS myelination (23, 31). GPR56 expression was highest in the oligodendrocyte precursor cells (OPC), but declined gradually during oligodendrocyte maturation and was very low, if not absent, in mature myelinating oligodendrocytes (23, 32). Importantly, disruption of the GPR56 gene reduced the proliferation ability of OPC leading to a defect of CNS myelination (23). It was found that GPR56 regulated the proliferation of OPC via the Gα12/13 and RhoA signaling pathways (31). Hence, Gpr56 deficiency prompts the premature cell cycle exit of OPC, leading to less mature oligodendrocytes (23, 31). In a more recent study, GPR56 was shown to be also involved in myelin development and maintenance in the peripheral nervous system by interacting with plectin (33).

Next to the CNS development, GPR56 also plays a role in other developmental processes such as muscle cell differentiation, male fertility and immune regulation. As such, upregulated GPR56 expression is detected during early myoblast differentiation. Importantly, GPR56 deletion causes primary myoblast cell abnormality *in vitro*, but muscle development *in vivo* was not affected (34). Moreover, a role in regulating muscle cell hypertrophy under resistance exercise conditions was noted recently for GPR56, which likely signaled via the Gα_12/13_ and mTOR pathways (35). In the reproductive system, GPR56 was involved in the testis development and male fertility as loss of GPR56 lead to reduced fertility in male mice due to partial disruption of seminiferous tubules. GPR56 was thought to be essential for the establishment of asymmetric testis cord remodeling during male gonad development (21). In the immune system, GPR56 is restrictedly expressed in cytotoxic lymphocytes including NK cells (36, 37). Subsequent investigation by us showed that GPR56 functions as an inhibitory receptor on human NK cells through cis-interaction with the tetraspanin CD81 (38).

Apart from the aforementioned biological and cellular functions, increasing evidences also implicate a critical role of GPR56 in tumor cell progression (39–41). Apart from the early description of its differential expression in melanoma cells, GPR56 expression was also detected in other cancer cell types including esophageal squamous cell (42), breast, non-small cell lung, ovarian, colon, pancreatic carcinoma (43, 44), and glioblastoma/astrocytoma (45). These studies indicated strongly a role of GPR56 in tumor cell adhesion, migration and survival/apoptosis (39–45). In addition, GPR56 is also strongly expressed in a subgroup of acute myeloid leukemia (AML) containing high levels of the ecotropic viral integration site-1 (EVI1) transcription factor (40). Interestingly, GPR56 gene is identified as a transcriptional target of EVI1 and increased GPR56 expression in EVI1-high AML promotes cell adhesion and anti-apoptotic activities (40). Furthermore, GPR56 is identified recently as a marker of human leukemia stem cells (LSCs), which give rise to AML. The GPR56 expression level in LSCs is positively correlated with high-risk AML diseases and poor clinical outcome. Engrafting GPR56-overexpressing AML cells into mice accelerates myeloid leukemogenesis and decreases the survival rate (40, 46, 47). Importantly, functional blockage of GPR56 by specific antibody efficiently attenuated AML cell engraftment suggesting GPR56 as a potential anti-leukemic target (40, 46, 47). In addition, GPR56 was found to have an inhibitory role in the mesenchymal differentiation and radioresistance of glioblastoma by negatively regulating the nuclear factor kappa B (NF-κB) signaling pathway (48). Despite these advances, the role of GPR56 in tumor progression was investigated in depth mostly in melanoma cells, which we will discuss in the later section.

## Ligands/binding partners of GPR56

**Figure 1:**
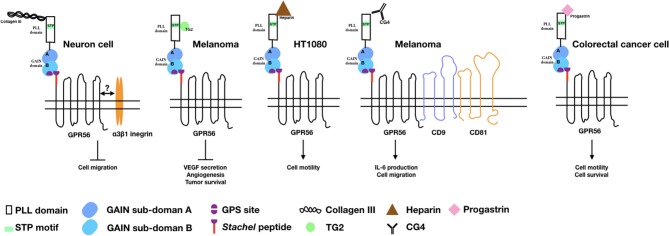
The binding partners/ligands of GPR56 and the cellular functions induced by the GPR56-ligand interaction. Diagrams depict the GPR56 receptor and its various binding partners/ligands as well as the cellular functions resulted from the GPR56-ligand interaction in relevant cell types as indicated.

As a canonical aGPCR, it is not surprising that several cellular ligands/binding partners have been identified for GPR56 in past years (Figure 1). The identification and functions of these cellular ligands/binding partners of GPR56 are discussed below.

## Collagen III

Collagen III (col-III), encoded by the Col3a1 gene, is expressed mostly in skin and blood vessel and is involved in the normal development of these tissues (49). While searching for a GPR56-specific ligand in brain, Piao's group found that GPR56-NTF interacted specifically with col-III which is highly expressed in the meninges, pial BM, and blood vessels during mouse embryonic development (50). The col-III binding site of GPR56 was located at a.a. 27–160 and several BFPP-associated point mutations including R38Q, R38W, Y88C, and C91S all lost the col-III binding ability (28). Collagen III-GPR56 interaction activates Gα_12/13_ and RhoA pathways and inhibits the migration of neural cells *in vitro* (50–52). Functional analysis of the Col3a1^−/−^ mice identified very similar phenotypes of brain malformation seen in the GPR56 null animals, including over migration of neurons and a cobblestone-like appearance (50). Therefore, it is concluded that col-III is a major GPR56-ligand in the developing brain and acts to regulate the normal lamination process of the cerebral cortex. A later study showed that col-III binding to GPR56 facilitates the separation and release of GPR56-NTF from the GPR56-CTF, which translocate from the non-lipid raft to the lipid raft membrane regions and induces RhoA activation (52).

## Tansglutaminase 2 (TG2)

In clinical observation, the expression level of GPR56 is correlated inversely to the metastatic ability of melanoma cells, suggesting an inhibitory role in melanoma metastasis (12, 18, 39). Xu's group first demonstrated the presence of a putative GPR56-ligand in the extracellular matrix (ECM) of lung, stomach, and tumor tissues using the GPR56-NTF as a probe. They subsequently identified transglutaminase 2 (TG2) as the ECM-ligand of GPR56 in melanoma tissues (39). It was found that the C-terminal region of TG2 interacts directly with the serine threonine proline-rich (STP) segment of the GPR56-NTF (39, 53). Importantly, it was shown that the binding of TG2 to GPR56 triggered the endocytosis process so that TG2 was internalized and degraded in the lysosome (54). This led to reduced deposition of fibronectin in the ECM and attenuated repertoire of focal adhesion kinase. These results indicated an antagonistic GPR56-TG2 relationship in regulating ECM deposition and cell-ECM adhesion during melanoma cell progression (39, 54). It is likely that TG2 is the dominant tissue environmental determinant regulating the metastatic potential of GPR56-expressing melanoma cells (55).

## Heparin

Besides the two protein ligands described above, heparin was verified by us recently as a novel glycosaminoglycan (GAG) binding partner of GPR56 (56). Interestingly, several aGPCRs including EMR2/ADGRE1, CD97/ADGRE5, and GPR124/ADGRA2 had also been shown to interact with distinct GAGs, suggesting a ubiquitous aGPCR-GAG partnership (57–59). The putative heparin-binding sites of GPR56 are mapped to two regions rich in basic residues at a.a. 26–35 and a.a. 190–200, which overlap partially with the col-III and TG2-binding sites. Most interestingly, both col-III and TG2 are also heparin-binding proteins, but only col-III is able to modulate the GPR56-heparin interaction (56). Unexpectedly, heparin binding attenuated GPR56-NTF shedding leading to enhanced cell adhesion and motility (56). This finding highlights the functional complexity of the interaction of GPR56 with its diverse endogenous ligands.

## Cis-interacting molecules

While the role of cellular ligands/binding partners that interact with GPR56 in-trans has been delineated, a couple of putative cis-interacting molecules of GPR56 have also been identified. Little at al. showed that GPR56 formed a complex with the tetraspanin CD81 and Gα_q/11_ from which at least two distinct activation mechanisms can be induced. It was concluded that CD81 probably acted as a GPCR-scaffolding protein that promotes and stabilizes the GPR56 signaling complex (60). Indeed, a role of CD81 in regulating GPR56 receptor function in human NK cell was verified by us recently (38). In addition, a functional role of a putative GPR56 accessory molecule, the α_3_β_1_ integrin, in brain development has also been demonstrated by Jeong et al. (51). Previous studies have shown that GPR56-deficient granule cells reduced its adhesion to laminin, a ligand of the α_3_β_1_ integrin that is also involved in the pial BM integrity (51). Interestingly, the cortical phenotype was exacerbated in the GPR56/α_3_-integrin double knock out animals. The results indicate strongly a synergistic collaboration between GPR56 and α3β1 integrin in regulating the cortical development (51). Intriguingly, most tetraspanin molecules such as CD9, CD81 and CD151, are known to associate with the integrin proteins on membrane (61–63). Hence, these data suggest the presence of a possible GPR56-tetraspanin-integrin super-complex that awaits further investigation.

## Progastrin

Progastrin, a growth factor and potent pro-angiogenic factor, was identified recently as a soluble ligand of GPR56 (41). Progestin has been known to enhance the proliferation of colonic epithelial cells and the progression of colorectal cancer in mice (64–66). Jin et al. showed that overexpression of human progastrin in mice stimulated GPR56 expression in colonic mucosa cells. Furthermore, progastrin was able to bind to cell surface GPR56 and the proliferation of GPR56-expressing colorectal cancer cells was increased in the presence of progastrin (41). Likewise, progastrin treatment enhanced the growth and survival of colonic organoids derived from the wild-type but not GPR56-null mice (41). Progastrin-dependent colonic proliferation *in vivo* was inhibited by the loss of GPR56. Finally, GPR56 deficiency also abrogated progastrin-dependent colonic crypt fission and colorectal carcinogenesis in a mouse model of colorectal cancer (41). Hence, the progastrin-GPR56 axis is thought as an important promoter of colorectal tumorigenesis *in vivo*.

## GPR56-specific Abs, monobodies, and small-molecule ligand

Over the years, the development of aGPCR-specific reagents such as antibodies (Abs) has facilitated the functional delineation of the receptors. Indeed, several GPR56-specific Abs have been produced to help dissect its activation mechanism and cellular functions. Iguchi et al. first detected strong GPR56 expression in neural progenitor cells (NPCs) using polyclonal Abs against the GPR56-NTF. GPR56 overexpression in NPCs was shown to negatively regulated cell migration. Importantly, they subsequently identified an agonistic activity of the GPR56-specific polyclonal Abs which were able to functionally inhibit NPC migration in a Gα_12/13_- and Rho-dependent manner (67). In recent years, we have generated five mouse monoclonal antibodies (mAbs) against human GPR56 (37). One of the mAbs, CG4, was found to activate GPR56 in melanoma cells when immobilized on the plate (12). Specifically, it was shown that interaction with the immobilized CG4 ligated and activated GPR56 on melanoma cells to enhance IL-6 secretion in a CD9/CD81-dependent manner via the Gα_12/13_- and Rho-signaling pathways (12). The mechanistic mechanism of CG4-induced GPR56 activation in melanoma cell involves the shedding of GPR56-NTF and will be discussed in detail in the later section.

Ohta et al. similarly generated several GPR56 mAbs, some of them were able to inhibit human U87-MG glioma cell migration via the Gα_q_- and Rho-signaling pathways. Interestingly, the agonistic mAbs were found to enhance NTF-CTF interaction as revealed by co-immunoprecipitation analysis (68). More recently, Salzman et al. succeed in selecting engineered monobodies with high specificity and affinity to the GPR56-NTF. These monobodies not only help stabilize and determine the crystal structure of the GPR56-NTF and the identification of the novel PLL domain, but also reveal a Stachel-independent NTF-mediated activation mechanism of GPR56 (16, 19). The effect of the GPR56-specific monobodies on cellular functions however remains unexplored. Finally, Stoveken et al. identified a small-molecule ligands of GPR56, dihyromunduletone (DHM) and 3-α-acetoxydihydrodeoxygedunin (3-α-DOG) which is antagonist and agonist respectively, from a library of compounds using a reporter-based screening strategy (69, 70). DHM and 3-α-DOG modulated GPR56 activation induced by the tethered as well as the exogenous synthetic Stachel peptides. Interestingly, DHM and 3-α-DOG also regulated the activation of GPR114/ADGRG5 apparently due to the highly similar Stachel peptide sequence shared with GPR56. Anyhow, DHM and 3-α-DOG represent small-molecule aGPCR ligands capable of modulating aGPCR activity.

## Alternative splice variants of GPR56

While most studies focused on the ligand-receptor interaction or the expression level of GPR56 during tumor progression, the composition of GPR56 protein variants might also influence the tumorigenesis. A total of five GPR56 receptor variants have been identified, including the full-length wild-type and four alternative splice variants (71). Variants 1 and 2 both missed eight amino acids present at the first intracellular loop, with the variant 2 contains an extra five residues at the cleavage site of the signal peptide. WT, variants 1 and 2 promote the downstream factors expression not only including the SRE response elements, but also COX2, iNOS, and VEGF which contributed to tumor growth (71). Both variants 3 and 4 exhibit a large deletion compared to the WT. Variant 3 contains a 170-residue deletion (38–208) which truncated the PLL domain (19). Translation of variant 4 starts with ATG at the exon 3 (19, 71). These results further confirmed a critical role of the extracellular PLL domain in regulating GPR56 function (19). Hence, it is of importance to consider the splice variants of GPR56 when examining its role in tumorigenesis.

## The activation and signaling mechanisms of GPR56/ADGRG1 in melanoma cell

The GPR56-mediated signaling pathways have been investigated by several groups in diverse cell types. These include the GPR56/CD81/Gα_q/11_ complex described in HEK293 and U937 cells by Little et al. (60) and the GPR56 polyclonal Ab-induced Gα_12/13_ and RhoA pathway in NPCs by Iguchi et al. (67). Subsequent studies on oligodendrocytes and other cell types also highlighted the involvement of the Gα_12/13_ and RhoA pathways (12, 31, 50, 72). Herein, we will focus on the activation and signaling mechanism of GPR56 in melanoma cells.

**Figure 2:**
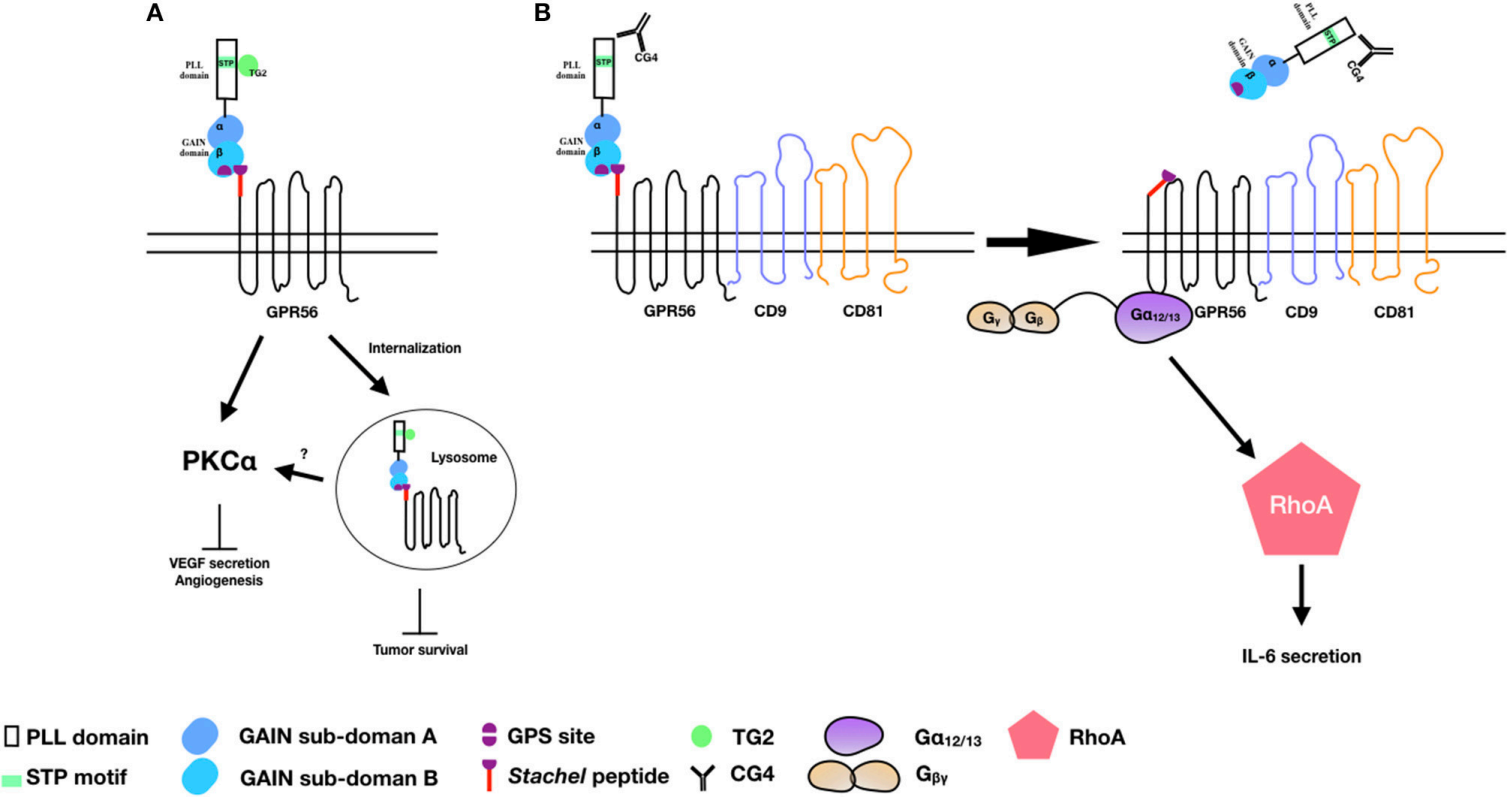
The activation and signaling pathways of GPR56 in melanoma cells. **(A)** TG2 binding induces GPR56 receptor internalization to inhibit melanoma growth and angiogenesis. It is also likely that GPR56 can signal via PKCα to inhibit VEGF production and angiogenesis through TG2 binding. **(B)** The CG4 mAb binds to GPR56 in the presence of CD9/CD81, causing NTF shedding and the subsequent activation of CTF by the Stachel peptide. The signaling is mediated through the Gα_12/13_ and RhoA pathways, leading to IL-6 production.

Consistent with the earlier report of reduced expression of GPR56 transcripts in highly metastatic melanoma cell lines, Xu et al. showed that GPR56 was significantly down-regulated in highly metastatic cells in an *in vivo* metastatic model of human melanoma cells (39). The inhibitory role of GPR56 in melanoma tumor growth and metastasis was further confirmed using specific RNAi-mediated gene silence. Indeed, TG2 was identified in this study as a potential ECM ligand that contributed to the metastatic suppressive function of GPR56. This is likely due to the antagonistic effect of TG2-GPR56 interaction on the deposition of ECM and ECM-cell adhesion as mentioned above (54). Furthermore, GPR56 was shown to suppress the angiogenesis and growth of melanoma by inhibiting the production of vascular endothelial growth factor (VEGF) via the PKCα signaling pathway in a STP domain-dependent manner (53). Because the STP domain is the TG2-binding region, these results implied that by binding to the STP domain, TG2 induced GPR56-mediated inhibition of VEGF secretion and angiogenesis via the inactivation of PKCα (Figure 2A) (53).

By contrast, we showed that through the binding and ligation of immobilized CG4 mAb, GPR56 receptor cross-linking in melanoma cells promoted the shedding of GPR56-NTF and subsequent activation and signaling of GPR56-CTF via the Gα_12/13_ and RhoA pathways, resulting in IL-6 production. Interestingly, the CG4-induced NTF shedding required the presence of CD9/CD81 as CD9/CD81 knockdown significantly reduced IL-6 secretion. It was further shown that the dissociation of GPR56-NTF unmasked the Stachel peptide of GPR56-CTF, which was responsible for activating CTF and the signaling transduction. Hence, our results are in line with the tethered agonist model of aGPCRs of which the receptor activation requires the release of the NTF of a fully GPS-cleaved receptor in a Stachel peptide-dependent manner (Figure 2B) (12).

While these studies provide important insights into the role of GPR56 in tumorigenesis, especially in melanoma progression, our understanding of GPR56 function in tumor biology is just at its infancy. For example, conflicting outcomes were observed in the involvement of GPR56 in the development of endogenous tumors (73). More recently, alternative GPS proteolysis-independent and Stachel-independent mechanisms of GPR56 activation have been uncovered. It is very likely that GPR56 is involved in different stages of tumor cell progression by multiple activation pathways.

## Conclusions and discussions

GPR56/ADGRG1 not only involves in the normal tissue development but also in tumorigenesis. Previous studies have identified numerous cellular binding partners/ligands and developed specific Abs/monobodies of GPR56. Meanwhile, distinct GPR56-mediated signaling pathways are being revealed. These unique reagents provide excellent research tools for the functional delineation of GPR56 and identify GPR56 as an important player in melanoma development. Functional studies with these GPR56-specific reagents including mAbs, monobodies, and small-molecule compounds have demonstrated effective modulation of its signaling activities (16, 46, 70). It is hoped that better understanding of GPR56-mediated activation and signaling mechanisms as well as the development of GPR56-sepcific reagents in the future may help target GPR56 as a potential cancer therapeutic molecule, especially in melanoma.

## Author contributions

K-YH and H-HL drafting and writing of the manuscript.

### Conflict of interest statement

The authors declare that the research was conducted in the absence of any commercial or financial relationships that could be construed as a potential conflict of interest.
